# Effect of neoadjuvant chemotherapy on tumor-infiltrating lymphocytes and PD-L1 expression in breast cancer and its clinical significance

**DOI:** 10.1186/s13058-017-0884-8

**Published:** 2017-08-07

**Authors:** Vasiliki Pelekanou, Daniel E. Carvajal-Hausdorf, Mehmet Altan, Brad Wasserman, Cristobal Carvajal-Hausdorf, Hallie Wimberly, Jason Brown, Donald Lannin, Lajos Pusztai, David L. Rimm

**Affiliations:** 1 Department of Pathology, Yale University School of Medicine, 310 Cedar St, PO Box 208023, New Haven, CT 06520-8023 USA; 2 Department of Medical Oncology, Yale University School of Medicine, New Haven, CT USA; 3 Department of Surgery, Yale University School of Medicine, New Haven, CT USA

**Keywords:** Tumor infiltrating lymphocytes, Programmed death ligand 1, Neoadjuvant treatment, Breast cancer

## Abstract

**Background:**

The effects of neoadjuvant chemotherapy on immune markers remain largely unknown. The specific aim of this study was to assess stromal tumor-infiltrating lymphocytes (TILs) and programmed death ligand 1 (PD-L1) protein expression in a cohort of breast cancer patients treated with neoadjuvant chemotherapy.

**Methods:**

Using quantitative immunofluorescence, we investigated stromal TILs and PD-L1 protein expression in pre-treatment﻿ and residual breast cancer tissue from a Yale Cancer Center patient cohort of 58 patients diagnosed with breast cancer from 2003 to 2009 and treated with neoadjuvant chemotherapy. We compared the TIL count and PD-L1 status in paired pre-treatment and residual cancer tissues and correlated changes and baseline levels with survival.

**Results:**

Of the 58 patients, 46 (79.3%) had hormone-positive and 34 (58.6%) had node-positive breast cancer. Eighty-six percent of residual cancer tissues had TIL infiltration and 17% had PD-L1 expression. There was a trend for higher TIL counts in postchemotherapy compared to prechemotherapy samples (*p* = 0.09). Increase in TIL count was associated with longer 5-year recurrence-free survival (*p* = 0.02, HR = 3.9, 95% CI = 1.179–15.39). PD-L1 expression (both stromal and tumor cells) was significantly lower in post-treatment samples (*p* = 0.001). Change in PD-L1 expression after therapy or TILs and PD-L1 expression in the posttreatment samples did not correlate with survival.

**Conclusions:**

Increase in stromal TILs in residual cancer compared to pretreatment tissue is associated with improved recurrence-free survival. Despite a trend for increasing TIL counts, PD-L1 expression decreased in residual disease compared to pretreatment samples.

**Electronic supplementary material:**

The online version of this article (doi:10.1186/s13058-017-0884-8) contains supplementary material, which is available to authorized users.

## Background

Neoadjuvant chemotherapy is increasingly used to induce tumor shrinkage, allowing smaller surgical resection, eliminating clinically silent micrometastases, and providing prognostic information based on the extent of pathologic response. Pathologic complete response (pCR) predicts excellent survival while residual disease (RD) is associated with higher but variable risk of recurrence depending on the molecular subtype [1–4]. Pretreatment immune infiltration in breast cancer predicts both for better prognosis, with or without adjuvant therapy, and also for greater sensitivity to chemotherapy reflected by the higher pCR rates in immune-rich cancers [5, 6].

Several preclinical studies have suggested that cytotoxic agents partly exert their anti-tumor activity by induction of an anti-tumor immune response aimed at cells injured by chemotherapy. Injury from chemotherapy may lead to formation of new immunogenic epitopes, cytokine secretion, antigen cross-presentation, activation of dendritic cells, and induction of tumor-specific cytotoxic T cells. Recent studies on breast cancer patients have suggested that cytotoxic agents, including anthracyclines and taxanes, can induce a tumor-specific immune response, and that exposure to such drugs leads to accumulation of lymphocytes in the tumor bed [7–9]. However, chemotherapy also has a direct cytotoxic effect on lymphocytes and could adversely impact the tumor immune microenvironment [10, 11].

The simplest measure of immune activity in the tumor microenvironment is counting tumor infiltration lymphocytes (TILs). Many studies have shown that TILs in the tumor microenvironment are prognostic, particularly for ER-negative and highly proliferative ER-positive cancers [12–14]. High TIL count is also associated with higher pCR rate after neoadjuvant chemotherapy [9, 12, 13, 15, 16]. High TIL count in residual disease is also associated with better survival [9–11].

The programmed cell death 1 receptor (PD-1) and its ligand PD-L1 are key immune regulatory molecules that play a pivotal role in switching off cytotoxic immune response as part of a complex immune checkpoint process [17]. PD-L1 expression is present in a variety of cancers including those of the lung, melanoma, ovarian, colon, and breast [14, 17–21]. PD-L1 is expressed by both tumor and stroma cells, and the tumor versus stromal expression frequency varies by cancer type. PD-L1 expression in the tumor microenvironment correlates strongly with the presence of TILs [14, 19, 20, 22, 23]. Drugs that target the PD-1 or PD-L1 axis have demonstrated durable tumor responses in 10–40% of patients in clinical trials including metastatic melanoma, renal cell carcinoma, nonsmall cell lung cancer, bladder cancer, triple-negative breast cancer (TNBC), and several other solid and hematological malignancies [24, 25].

The goal of this study was to assess changes in TIL count and PD-L1 expression in response to neoadjuvant chemotherapy for early-stage breast cancer. We reported previously on the prognostic and chemotherapy response predictive value of pretreatment TIL count, PD-L1 expression, and Ki-67 in the same patient cohort [14–16, 20]. The current analysis includes examination of TILs and PD-L1 expression in the residual tumor and we assess changes between paired pre-treatment and post-treatment samples.

## Methods

### Patient cohort

Pre-treatment and post-treatment formalin-fixed paraffin-embedded breast cancer tissues were retrieved from the archives of the Department of Pathology at Yale University (New Haven, CT, USA). Patients were diagnosed between 2002 and 2010. Fifty-eight patients with residual disease who had received neoadjuvant chemotherapy, and for whom tissue was also available from baseline specimens, were included in this study. Cases with pCR were not included in this study. Fifty-six percent of patients received four cycles of doxorubicin and cyclophosphamide followed by four cycles of taxane, and the rest received various other chemotherapy regimens (detailed in Additional file 1: Table S1). The distribution of treatment regimens, grade, tumor size, hormone receptor status, and HER2 status is presented in Table 1. Relapse-free survival (RFS) and overall survival (OS) data were available for 34 and 57 patients, respectively. We defined relapse as including all kinds of relapse (local, distant, ipsilateral, contralateral). This retrospective research and tissue collection was reviewed and approved by the Yale Human Investigation Committee protocol #9505008219 and/or #1010007459 prior to collection. The Yale Human Investigation Committee approved the patient consent forms (including publication of research data) or in some cases a waiver of consent. Images from tissue specimens are entirely unidentifiable and there are no details on individuals reported within the manuscript.

**Table 1:** Clinicopathological characteristics of patients

Characteristic	*N* (%)
Total	58 (100)
Pre-neoadjuvant TIL evaluation available	43 (74.1)
Post-neoadjuvant TIL evaluation available	58 (100)
Age (range 28–71 years)	
<50 years	28 (48.2)
≥50 years	30 (51.7)
Histotype	
Ductal	50 (86)
Other	8 (3)
DCIS foci	8 (13.7)
ER	
Negative	12 (20.6)
Positive	45 (77.5)
TNBC	9 (15.5)
PR	
Negative	17 (29.3)
Positive	40 (68.9)
HER2	
Negative	46 (79.3)
Positive	12 (20.6)
Lymph node status	
Negative	21 (36.2)
Positive	37 (63.7)
Residual tumor size	
<2 cm	24 (41.3)
> 2 cm	34 (58.6)
Grade at diagnosis	
1 and 2	37 (63.7)
3	19 (32.75)
N/A	2 (0.034)
Relapse	
No relapse	25 (43.1)
Relapse	11 (18.9)
N/A	22 (37.9)
Neoadjuvant chemotherapy	
AC/taxane	33 (56.8)
Other	25 (43.1)

*TIL* tumor infiltrating lymphocyte, *DCIS* ductal carcinoma in situ, *ER* estrogen receptor, *TNBC* triple-negative breast cancer, *PR* progesterone receptor, *HER* human epidermal growth factor, *AC* anthracycline, *N/A* not available

### Pathology evaluation of TILs

Hematoxylin and eosin-stained (HES) slides from pretreatment core biopsy and post-neoadjuvant resection specimens were scored for stromal TILs based on Immuno-Oncology Biomarker Working Group guidelines [12, 26]. Stromal TIL scores were defined as the percentage of tumor stroma area that was occupied by mononuclear inflammatory cells. TILs were scored as continuous variables with positivity cutoff set at 1%. Stroma was evaluated only in slides with invasive tumor. Inflammatory infiltrates in the stroma of noninvasive lesions (including DCIS) and normal breast structures were excluded. Slides from matched pretreatment biopsies were also assessed. Change in TIL was defined as the difference (TIL counts post treatment – TIL counts pre treatment). Although the guidelines for TIL evaluation have been established on baseline tumors, they have also been used by previous studies of residual disease [9, 27].

### Antibodies and immunofluorescent staining

Freshly cut whole-tissue sections of post-treatment specimens and a technical control tissue microarray (TMA) slide were baked overnight at 60 °C and then soaked in xylene twice for 20 minutes each. Slides were rehydrated in two 1-minute washes in 100% ethanol followed by one wash in 70% ethanol and finally rinsed in streaming tap water for 5 minutes. Antigen retrieval was performed in EDTA, pH 8, in the PT module from LabVision (Thermo Scientific, Waltham, MA, USA). Endogenous peroxidases were blocked by 30-minute incubation in 2.5% hydrogen peroxide in methanol. Subsequent steps were carried out on the LabVision 720 Autostainer (Thermo-Scientific, Waltham, MA, USA). Nonspecific antigens were blocked by 30-minute incubation in 0.3% BSA in TBST. Primary PD-L1 (SP142) rabbit monoclonal antibody (Spring Bioscience; see Additional file 2: Figure S1 for antibody validation) was prepared to a working concentration of 0.154 μg/ml combined with 1:100 pan-cytokeratin (AE1/E3) antibody (Dako) in 0.3% BSA in TBST and transferred to 4 °C overnight. Primary antibodies were followed by incubation with Alexa 546-conjugated goat anti-mouse secondary antibody (Molecular Probes, Eugene, OR, USA) diluted 1:100 in rabbit EnVision reagent (Dako) for 1 hour. The signal was amplified with Cyanine 5 (Cy5) directly conjugated to tyramide (Perkin-Elmer, Waltham, MA, USA) at 1:50 dilution was used for target antibody detection. ProLong mounting medium (ProLong Gold; Molecular Probes) with 4,6-diamidino-2-phenylindole (DAPI) was used to stain nuclei.

Pre-treatment samples were stained with a different anti-PD-L1 rabbit monoclonal antibody (clone E1L3N; Cell Signaling Technology) as described previously [20]. We have also reported previously on the concordance of results after testing both antibodies on the same tissues [18, 28].

### Fluorescent measurement and scoring

Quantitative immunofluorescence (QIF) was performed using the AQUA method [29, 30] on freshly stained slides. Tumor and stromal compartments were defined as the area of cytokeratin positivity and the area of DAPI positivity after cytokeratin subtraction, respectively. Areas of normal, benign, or DCIS counterpart have been excluded from scoring. QIF scores for PD-L1 in the tumor and stromal compartment were calculated by dividing the target compartment pixel intensities by the area of the corresponding mask. QIF scores were normalized to the exposure time and bit depth at which the images were captured, allowing scores collected at different exposure times to be comparable. All acquired fields of view (range: 5–93, mean: 32) were evaluated visually and cases with staining artifacts or less than 1% invasive tumor (cytokeratin staining) were excluded from the analysis.

### Statistical analysis

A QIF score of 500 AU was used to stratify PD-L1 SP142 protein scores into positive or negative categories for analysis. This threshold was derived from visual inspection of all fields of view of breast cancer specimens and adequate control tissues (placenta and lung cancer) and cell lines. Protein levels, reflected by QIF scores as a continuous variable, were compared using linear regression coefficients (*R* ^2^) and the Mann–Whitney test. TILs and PD-L1 expression across patient subsets were compared using the χ^2^ test. Survival functions were compared using Kaplan–Meier curves, and statistical significance was determined using the log-rank test. The small size of the cohort precluded a multivariate analysis of survival. For the comparison of PD-L1 protein levels before (clone E1L3N; Cell Signaling Technology) and after (clone SP142; Spring Bioscience) neoadjuvant chemotherapy, we normalized using the maximum scores per assay. The highest PD-L1 E1L3N score was divided by the highest PD-L1 SP142 score. The quotient was used to divide all E1L3N values, and scale them to SP142. Statistical analysis was carried out using JMP 11.0 and GraphPad Prism v6.0 software (GraphPad Software, Inc., La Jolla, CA, USA). All *p* values were based on two-sided tests, and *p* < 0.05 was considered statistically significant and were not adjusted for multiple comparisons.

## Results

### TIL count in residual cancer specimens

**Fig. 1:**
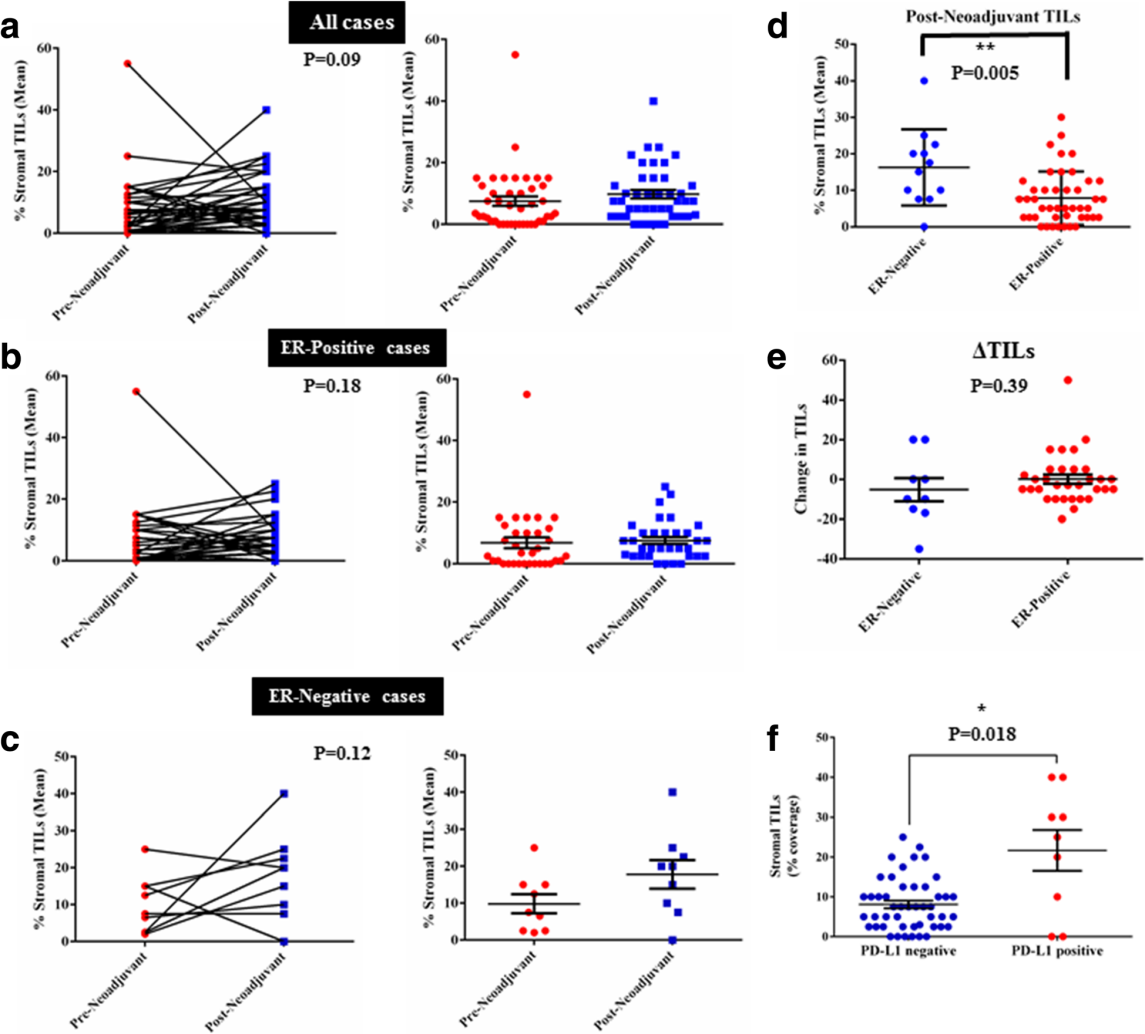
Stromal tumor-infiltrating lymphocyte (*TIL*) changes prior to (*pre-neoadjuvant*) and following (*post-neoadjuvant*) neoadjuvant chemotherapy. **a** Changes from diagnostic core biopsy (pre-chemotherapy) to surgical specimen (post-chemotherapy) in all cases. *Right*: comparison of the two groups by Mann–Whitney test (mean and SEM, *p* = 0.09). Similar representation of changes before and after chemotherapy in estrogen receptor (*ER*)-positive cases (**b**) and ER-negative cases (**c**). **d** TIL count in residual tumor specimens in ER-negative and ER-positive cases is significantly different (mean and SEM shown). **e** Changes from pre-neoadjuvant to post-neoadjuvant (ΔTILs) display no significant difference in ER-positive and ER-negative tumors (mean and SEM, Mann–Whitney test). **f** Average stromal TIL percentage in residual tumor specimens (post-neoadjuvant) in relation to programmed death ligand 1 (*PD-L1*) status (Mann–Whitney test)

TILs were evaluated in 58/58 (100%) residual cancer cases. TILs were present in 50 of 58 (86.2%) residual cancer specimens. In most of these cases (29/37, 78%) TILs were also present in the matching pretreatment samples (Fig. 1). We did not assess the residual tumor bed in cases with pCR because these patients were not included in our study cohort. TILs were counted by two independent pathologists and the interobserver correlation coefficient was 0.9. As observed previously, lymphocyte predominance was rare; only one case had >50% stromal TILs. From 58 cases of residual disease, based on tissue and HES availability, TILs were scored in 43/58 (74.1%) matching baseline biopsies. The median TIL counts in pre-treatment and post-treatment specimens were 5% (range 0–60%) and 7.5% (range 0–40%), respectively. The median difference of absolute TIL (ΔTIL) count in pre-treatment compared to post-treatment specimens was 5% (range 0–50%), indicating an overall increase in TILs in residual disease samples compared to baseline. However, the post-treatment increase in TIL counts did not reach statistical significance (*p* = 0.09). In ER-negative tumors, the median post-treatment TIL count was 12.5% (range 0–40%), while in ER-positive tumors the median TILs count was 5% (range 0–30%) consistent with previously reported generally higher TILs in ER-negative cancers (Fig. 1a, b, c, respectively). Post-neoadjuvant TIL count was significantly higher in ER-negative cases (*p* = 0.005, Fig. 1d). Representative images of (HES) slides from pre-treatment core biopsies and post-neoadjuvant resection specimens are provided in Additional file 2: Figure S1.

**Fig. 2:**
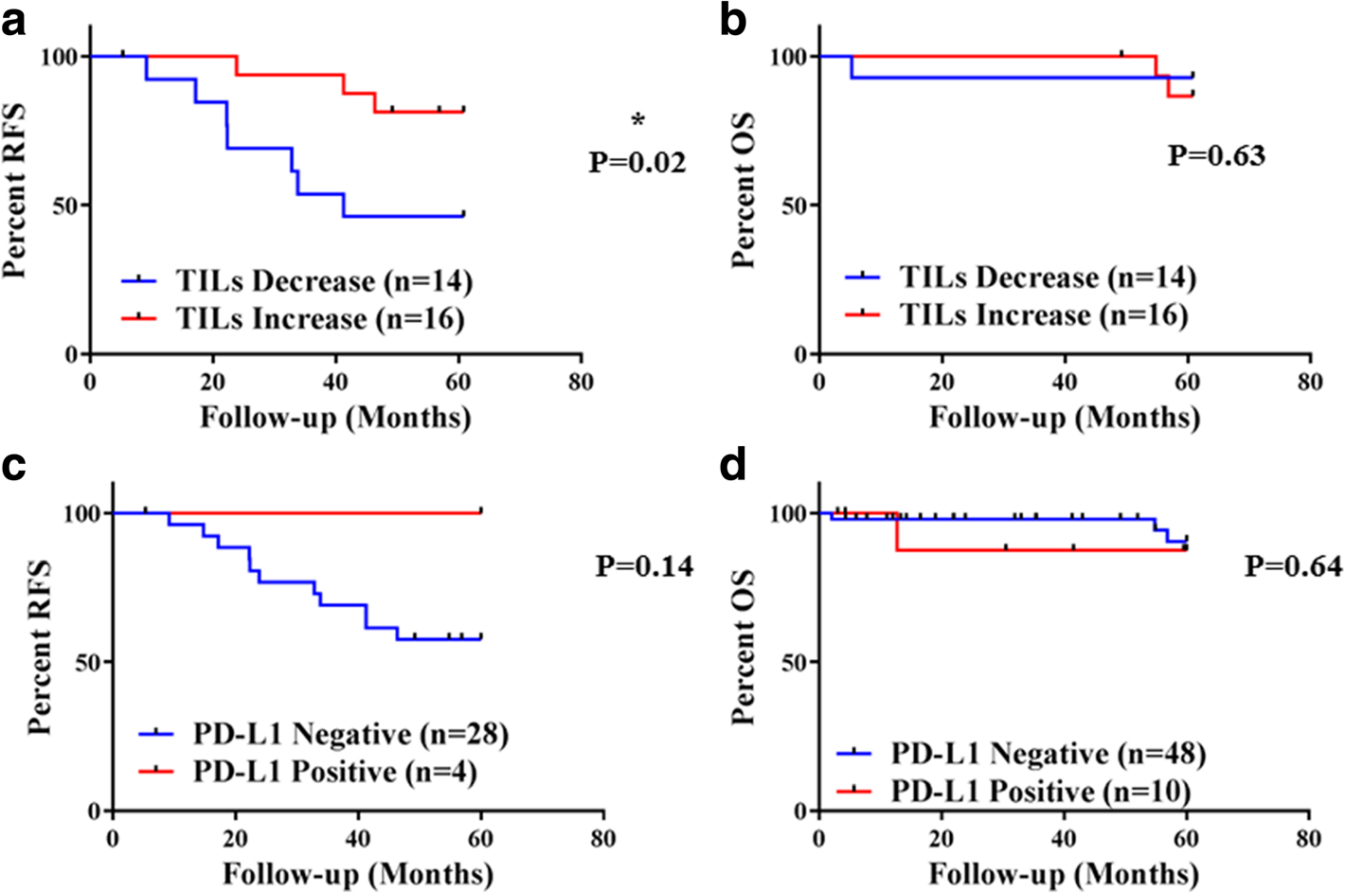
Comparison of PD-L1 expression prior to (*pre-neoadjuvant*) and following (*post-neoadjuvant*) neoadjuvant treatment. Changes in PD-L1 QIF scores in (**a**) the tumor compartment and (**b**) the stromal compartment from pre-chemotherapy biopsy to surgical specimen (Mann–Whitney test, mean and SEM). **c** Representative QIF images (400× magnification, insert 100×) of matched pre-neoadjuvant (*left column*) and post-neoadjuvant (*right column*). *Upper row*: case with high PD-L1 expression in chemo-naive biopsy that displays low PD-L1 following treatment. *Middle row*: case with low PD-L1 expression prior to and increased expression in the residual tumor. *Lower row*: case with low PD-L1 expression before and after treatment. DAPI counterstaining in *blue*, cytokeratin tumor mask in *green*, and PD-L1 in *red*. **d** Distribution of PD-L1 expression in the stroma and tumor in 41 matched cases with pre-neoadjuvant and post-neoadjuvant specimens. Colors correspond to compartment (*red* for tumor and stroma, *green* for tumor only, *purple* for stroma only, and *blue* for PD-L1 negative cases) and *numbers in the column* show the number of cases for each condition. *PD-L1* programmed death ligand 1, *DAPI* 4,6-diamidino-2-phenylindole (*Color* figure online)

There was no significant association between TIL status (present vs absent) in residual cancer and survival (Fig. 2). However, an increase in TILs in the residual cancer compared to baseline was associated with significantly improved RFS (log-rank *p* = 0.02, HR = 3.9, 95% CI = 1.17–15.39) (Fig. 2). TIL evaluation in baseline biopsies and TIL count change following treatment were possible in 43/58 cases (Table 1). RFS and OS data were available for only 31/43 matched cases. TIL count as a continuous variable in residual cancer had no significant association with OS or RFS (Additional file 3: Table S2).

### PD-L1 expression in residual cancer specimens

**Fig. 3:**
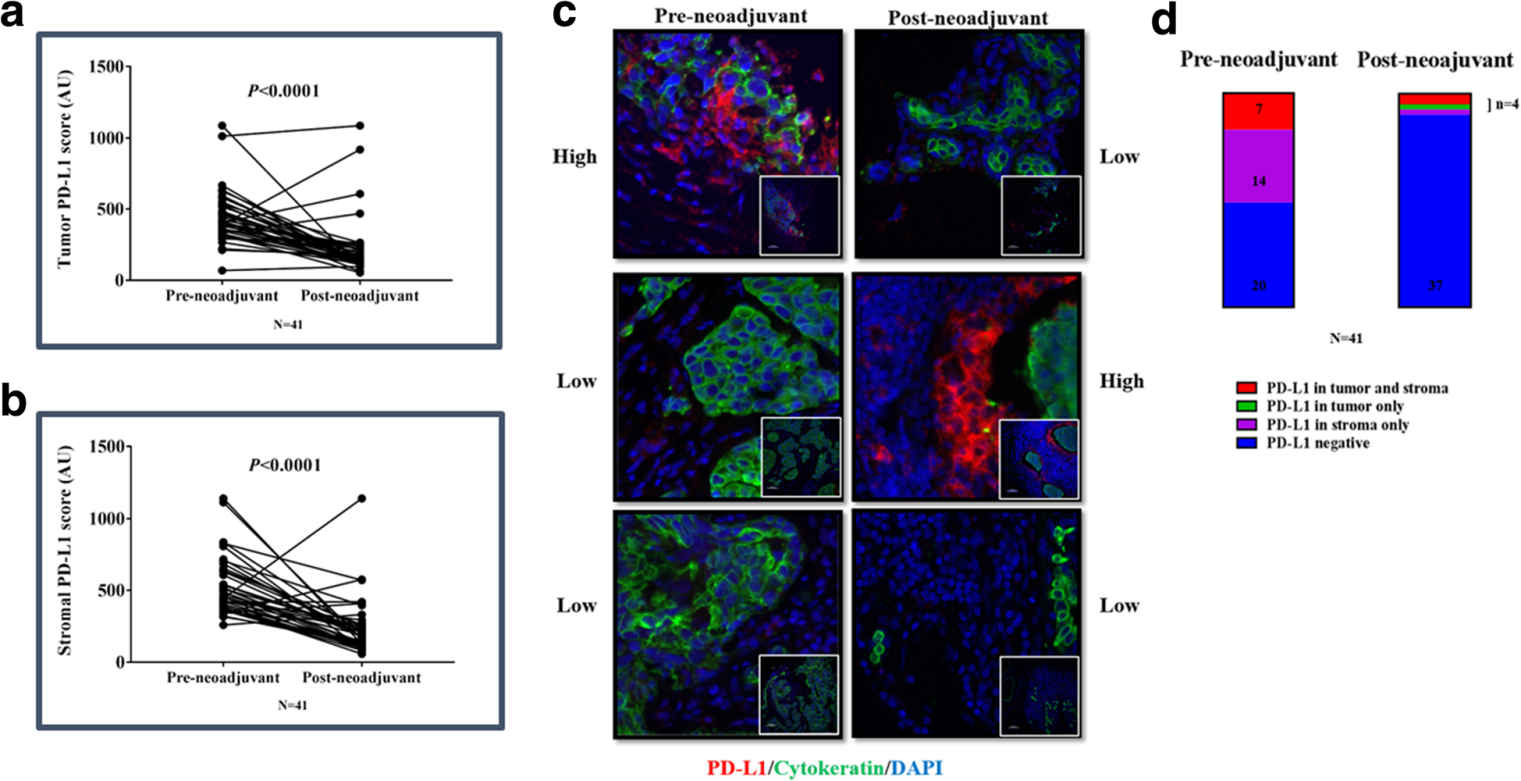
Survival analysis of patients with residual disease following post-neoadjuvant treatment based on ΔTILs and PD-L1 expression. **a** Five-year recurrence-free survival (*RFS*) Kaplan–Meier curve of stromal TIL change (increase or decrease) following neoadjuvant treatment. **b** Five-year overall survival (*OS*) Kaplan–Meier curve of stromal TIL change (increase or decrease) following neoadjuvant treatment. **c** Five-year RFS Kaplan–Meier curve of PD-L1 status (positive or negative) in post-neoadjuvant cases. Positivity based on the visual cutoff of 500 AQUA method units. **d** Five-year OS Kaplan–Meier curve of PD-L1 status (positive or negative) in post-neoadjuvant cases. *TIL* tumor infiltrating lymphocyte, *PD-L1* programmed death ligand 1

PD-L1 immunostaining was performed in 58/58 (100%) of residual tumor cases and in 41/58 (70.6%) of matching baseline biopsies. Missing cases were due to tissue availability/exhaustion or quality of staining. PD-L1 expression in the tumor and/or stroma was observed in 10 out of 58 (17.2%) residual cancer specimens using the QIF = 500 positivity threshold (Additional file 4: Figure S2A). PD-L1 expression was observed on both neoplastic and stromal cells; overall expression was higher in stromal cells than in neoplastic cells but there was a positive correlation between stromal and tumor PD-L1 levels (*R* ^2^ = 0.6, Additional file 4: Figure S2B). TIL and PD-L1 expression also correlated; stromal TILs were significantly higher in the PD-L1-positive cases (*p* = 0.018) (Fig. 1f). In univariate analysis, PD-L1 positivity was significantly higher in women < 50 years of age (*p* = 0.027) and residual tumor size < 2 cm (*p* = 0.044) (Table 2). In baseline biopsies, 21/41 (51.02%) cases were PD-L1-positive. Within the 41 matched baseline biopsies, 7/41 (17%) had PD-L1 expression in both tumor and stroma, 14/41 (34.14%) in stroma only, and 20/41 (48.7%) had no evidence of a specific signal. No case was identified with exclusively tumor PD-L1 expression at baseline. Comparison of PD-L1 QIF scores prior to and after neoadjuvant chemotherapy showed a significant decrease in the residual tumors (*p* < 0.0001, Fig. 2a, b). Following treatment, two cases remained positive in the tumor and stroma, and one in the tumor only. From the cases with exclusively stromal staining at baseline, only one was positive post treatment. Seventeen cases changed their PD-L1 status from positive to negative, and no cases became positive with treatment (Fig. 3a, d). PD-L1 protein expression in the residual cancer was not associated with survival, either as a categorical variable based on the positivity cutoff (Fig. 2) or as a continuous variable (Additional file 3: Table S2). Data on SP142 validation and assay reproducibility are provided in Additional file 5: Figure S3.

**Table 2:** Relationship between TILs, PD-L1, and clinicopathological characteristics in post-neoadjuvant breast cancer cases

	TIL status			PD-L1 status		
	Positive (*N* = 50)	Negative (*N* = 8)	*p* value	Positive (*N* = 10)	Negative (*N* = 48)	*p* value
Age						
< 50 years	22	6	0.103	8	20	0.027^a^
>50 years	28	2		2	28	
Size at surgery						
< 2 cm	19	5	0.191	7	17	0.043^a^
> 2 cm	31	3		3	31	
Histotype						
Ductal	43	7	0.909	8	42	0.53
Other	7	1		2	6	
Grade						
1 and 2	32	6	0.697	3	33	0.45
3	20	2		6	15	
Lymph node status						
Positive	32	5	0.934	8	29	0.241
Negative	18	3		2	19	
Hormone receptor status						
Positive	39	7	0.599	8	38	0.497
Negative	10	1		1	10	
HER2 status						
Positive	10	2	0.745	2	10	0.952
Negative	40	6		8	38	
Relapse status						
Relapse	9	2	0.37	11	0	0.159
No relapse	23	2		21	4	
N/A	27	4		16	6	
ΔTILs (pre–post)						
Positive or zero	15	5	**0.01**	19	1	0.206
Negative	23	0		19	4	

*TIL* tumor infiltrating lymphocyte, *PD-L1* programmed death ligand 1, *HER* human epidermal growth factor, *N/A* not available

^a^Significant (*p* < 0.05)

## Discussion

Several studies have examined the prognostic and chemotherapy response predictive role of TILs in baseline, pretreatment biopsies of breast cancer [12, 13, 16, 31–34], but few studies have examined change in TILs and immunological parameters during neoadjuvant therapy. In this study, we assessed changes in TIL count and PD-L1 expression in paired pre-neoadjuvant and post-neoadjuvant chemotherapy tissues and correlated residual cancer TIL counts and PD-L1 expression and change in these parameters with survival. We achieved a high interobserver concordance on TIL assessment which was based on the methodology of and recommendations by the Immuno-Oncology Biomarker Working Group [26, 33].

The effects of neoadjuvant chemotherapy on TILs and immune gene signatures have been mostly studied in TNBC and HER2-positive tumors [9–11, 13, 34, 35]. High TILs in residual cancer were associated with better RFS in two previous studies [9, 34]. We did not observe this in our study which may be due to our smaller sample size. On the other hand, we did observe that an increase of TILs in the residual cancer was associated with better RFS, confirming a prognostic role of TILs. The importance of change of TIL counts following neoadjuvant chemotherapy was also evidenced in a parallel study by our group [36] in specimens from the SWOG S0800 clinical trial [28]. Therein, we observed a significant decrease of TILs following treatment. Higher TIL change correlated with higher rates of pCR. The cohorts from the two studies are not comparable, as SWOG S0800 patients were HER-2 negative, enriched in TNBC and lymphocytic predominant breast cancer (9%), and under more homogeneous treatment. Hence, in both studies the TIL count change is indicative of better outcome. Further TIL subtyping is warranted to elucidate the functional status of lymphocytes involved.

Moreover, we have shown that TIL counts in residual disease were higher in ER-negative cases, but we were not able to correlate the TIL changes with ER status (increase, decrease, or extent of change). This could be due to the small size of our cohort. Interestingly, in the TRYPHAENA study [37] that compared TILs pre- and post- treatment with dual HER2 blockade, there was a significant overlap between ER-positive and ER-negative cases with residual disease. Similarly, there was a significant overlap between cases with low to moderate TIL infiltrate and achievement of pCR or not. Although this cohort is not directly comparable with ours, the results present some similarity in the difficulty to trace a clear trend between ER status and TIL change following treatment.

Our study is the first to report on changes in PD-L1 expression after neoadjuvant chemotherapy. Preclinical studies suggested that PD-L1 expression might be stimulated by chemotherapy [38]. However, in our study, only 17% of residual cancers were positive for PD-L1 expression using our AQUA method. The majority of cases were negative at baseline and remained negative after chemotherapy. In most of the initially PD-L1 positive tumors, PD-L1 expression decreased after chemotherapy. Therefore, overall, we observed a significant decrease in PD-L1 expression (as defined by continuous AQUA QIF scores) in residual disease compared to pretreatment biopsies. However, PD-L1 levels or changes in PD-L1 expression were not significantly associated with survival, but we cannot exclude the lack of power of our study to evaluate this. The neoadjuvant chemotherapy-induced changes in PD-L1 expression could provide a rationale for use of immune checkpoint inhibitors in the adjuvant and, most importantly, neoadjuvant setting. The results of the I-SPY2 [39] study showed important improvement of pCR by combined PD-axis blockade and chemotherapy in the neoadjuvant setting in breast cancer.

We also need to note that most PD-L1 expression was stromal. Although we did not use a PD-L1 multiplexed assay to characterize the cell types expressing PD-L1, we can assume based on morphology criteria that most PD-L1-expressing cells were not TILs, but more compatible with macrophages or fibroblasts. This observation could also explain why we observe this “disconnection” in PD-L1 and TIL change following treatment.

The field of PD-L1 assessment is rapidly evolving and several companion and complementary diagnostic applications are FDA cleared. In the clinic, all of the assays are based on DAB-based chromogen visualization. As a result, they lack standardization, and are limited by the subjective nature of this technique. Recently we have investigated the concordance of QIF and chromogenic assays (scored by pathologists) as well as the concordance between pathologists [40]. Pathologists were highly concordant for PD-L1 lung tumor scoring, but not for stromal/immune cell scoring. A similar concordance amongst pathologists for tumor PD-L1, but not immune cell PD-L1, was also described recently by the NCCN/BMS study on this topic [41]. In the case of breast cancer, the relevance of these findings might be even more impactful, as the levels of PD-L1 expression are much lower than in the case of lung cancer (usually less than 30%) [14, 20, 23] and, as we show here, PD-L1 is mainly stromal. A cutoff of 50%, like the one used in the 22c3 antibody chromogenic assay, would automatically exclude nearly all breast cancer cases from PD-1/PD-L1-based treatments. It is possible that other assays may be required to match PD-1 axis therapies to responders in this disease.

It is inherent to studies that compare baseline and residual breast cancer tissues after neoadjuvant chemotherapy that the tissue acquisition methods differ between pre- and post-treatment. The baseline tissues come from core needle biopsies, while the residual cancer tissues come from surgically resected tissue. This may introduce sampling bias; however, we previously examined intratumor heterogeneity in immunological parameters in primary breast cancers and found only modest biopsy site-to-site variation, and therefore the sampling bias may be less important in breast cancer than in other cancers [18, 42]. Because the pre-treatment PD-L1 assessment was also performed earlier, different antibodies were used to quantify PD-L1 expression in the pre-treatment (E1L3N) and post-treatment (SP142) tissues. While it would have been optimal to use the same antibodies for each aspect of the study, the timing of different parts of the study made this impossible. However, while the assays in the clinic are clearly different, the antibodies within the assays have been shown to be essentially identical [43]. The work by Gaule et al. from our group showed that, at optimal titration in optimal staining conditions, SP142 and E1L3N are nearly identical. Although the use of different antibodies is a limitation of this work, we believe our previous data make these results scientifically sound.

Another important limitation is the lack of specific guidelines in the assessment of TILs in residual disease. The definition of residual cancer burden, the inclusion of cases with pCR or not, and the evaluation of areas with invasive tumor only or in the “previous tumor bed” remain elusive. The guidelines of the Immuno-Oncology Biomarker Working Group have contributed in reproducibility of TIL evaluation among different studies, but they are not currently addressing these points. An updated version of TIL guidelines from the Immuno-Oncology Biomarker Working Group will include suggestions for TIL evaluation in the residual disease, later this year.

A more significant limitation of our study is that it is based on a small, single breast cancer cohort, with heterogeneity of breast cancer subtypes and non-uniform treatment administration. The tissues were collected retrospectively and included patients in different clinical stages and hormone receptor and HER2 status. Survival and disease status were also not available for all cases. The small sample size limited the statistical power to perform between subtype comparisons and adequately powered multivariate analysis. Larger, prospective studies, incorporating multi-institution cohorts, homogeneous breast cancer tumor subtypes, and treatment regimens, are required to validate and support the clinical relevance of our findings.

In summary, we observed a non-significant trend toward increased TIL counts in residual cancer tissues, whereas PD-L1 expression decreased. An increase in TIL count in residual cancer indicates more favorable RFS compared to no change or a drop in TIL counts.

## Conclusions

We have shown in patients who did not achieve pCR following neoadjuvant chemotherapy that even minor changes in TIL counts can provide hints for a better outcome. Importantly, this immune-related effect of neoadjuvant chemotherapy was observed in tumors that at baseline had low to moderate TIL counts, providing hints to further explore and valorize the functional status of immune signatures for these patients with residual disease.

Moreover, by QIF we have observed a significant decrease of PD-L1 expression following neoadjuvant chemotherapy. This disconnection with TIL counts is intriguing and could, at least partially, be explained by expression of PD-L1 by stromal cells, other than TILs, such as tumor-associated macrophages. Moreover, immune checkpoint blockade in the neoadjuvant setting could further enhance the effects of the conventional neoadjuvant chemotherapy alone.

Taken together, our data suggest the importance of further exploration of the immune potential of residual tumors in larger studies in order to ameliorate patients’ outcome by combined personalized immunotherapies. Inclusion of immunotherapy regimens in the neoadjuvant setting could also potentiate the pCR rates through multiple immune-parameter modulation. Hence, comprehensive host, tumor-intrinsic and microenvironmental baseline biomarker assessment is critical to predict benefit from personalized immunotherapies.

## Additional files

Additional file 1: Table S1. (XLSX 23 kb)

Additional file 2: Figure S1. Showing HES of TILs at baseline and post-treatment. **A** Baseline HES of a case with moderate TIL infiltration at baseline and increased TIL counts following treatment. **B** Matched post-neoadjuvant HES of the baseline biopsy shown in (**A**). **C** Baseline HES of a case that displayed decrease TIL counts following treatment. **D**. Matched post-neoadjuvant HES of the baseline biopsy shown in (**C**). 20× Magnification, *bar* = 200 μm. (TIF 948 kb)

Additional file 3: Table S2. Presenting logistic regression of TIL percentage and PD-L1 scores with survival. (DOCX 14 kb)

Additional file 4: Figure S2. Showing PD-L1 expression in post-neoadjuvant breast cancer specimens. **A** Distribution of maximal scores of PD-L1 (SP142 antibody) in the tumor (*red*) and stromal (*blue*) compartments. The cutoff was set at 500 AQUA units (QIF). **B** Linear regression of stromal versus tumor PD-L1 AQUA (QIF) scores. (TIF 148 kb)

Additional file 5: Figure S3. Showing SP142 antibody validation and reproducibility. **A** Regressions in QIF scores (average) between staining performed in different days in serial sections of a lung cancer TMA (245). **B** Representative immunostaining for PD-L1 SP142 antibody in control tissues (placenta in *upper panel* and lung in *lower panel*) using QIF (*left panel*; SP142 in Cy5, *red* and cytokeratin mask in Cy3, *green*) and conventional IHC staining with DAB (*right panel*). (TIF 583 kb)

## Abbreviations

AU: Arbitrary units
BSA: Bovine serum albumin
CI: Confidence interval
Cy5: Cyanine 5
DAPI: 4,6-Diamidino-2-phenylindole
DCIS: Ductal carcinoma in situ
ER: Estrogen receptor
HES: Hematoxylin and eosin-stained
HR: Hazard ratio
NCCN/BMS: National Comprehensive Cancer Network/Bristol-Myers-Squibb
OS: Overall survival
pCR: Pathologic complete response
PD-L1: Programmed death ligand 1
QIF: Quantitative immunofluorescence
RD: Residual disease
RFS: Relapse-free survival
SEM: Standard error of Mean
TBS: Tris-buffered saline
TBST: Tris-buffered saline (TBS) and Polysorbate 20 (also known as Tween 20)
TIL: Tumor infiltrating lymphocyte
TMA: Tissue microarray
TNBC: Triple-negative breast cancer
ΔTIL: Difference of absolute TIL count in pre compared to post treatment
