# Erratum to: Effect of neoadjuvant chemotherapy on tumor-infiltrating lymphocytes and PD-L1 expression in breast cancer and its clinical significance

**DOI:** 10.1186/s13058-017-0898-2

**Published:** 2017-09-25

**Authors:** Vasiliki Pelekanou, Daniel E. Carvajal-Hausdorf, Mehmet Altan, Brad Wasserman, Cristobal Carvajal-Hausdorf, Hallie Wimberly, Jason Brown, Donald Lannin, Lajos Pusztai, David L. Rimm

**Affiliations:** 1 Department of Pathology, Yale University School of Medicine, 310 Cedar St, PO Box 208023, New Haven, CT 06520-8023 USA; 2 Department of Medical Oncology, Yale University School of Medicine, New Haven, CT USA; 3 Department of Surgery, Yale University School of Medicine, New Haven, CT USA

## Erratum

The original article [1] has been updated. When originally published, the legends for Fig. 2 and Fig. 3 were in the wrong order, they have been switched back to the correct Figure.
