# Supplementary figures and images for: Effect of neoadjuvant chemotherapy on tumor-infiltrating lymphocytes and PD-L1 expression in breast cancer and its clinical significance
Source: Breast Cancer Res. 2017 Aug 7;19:91. doi: 10.1186/s13058-017-0884-8 (PMC5547502; doi:10.1186/s13058-017-0884-8)

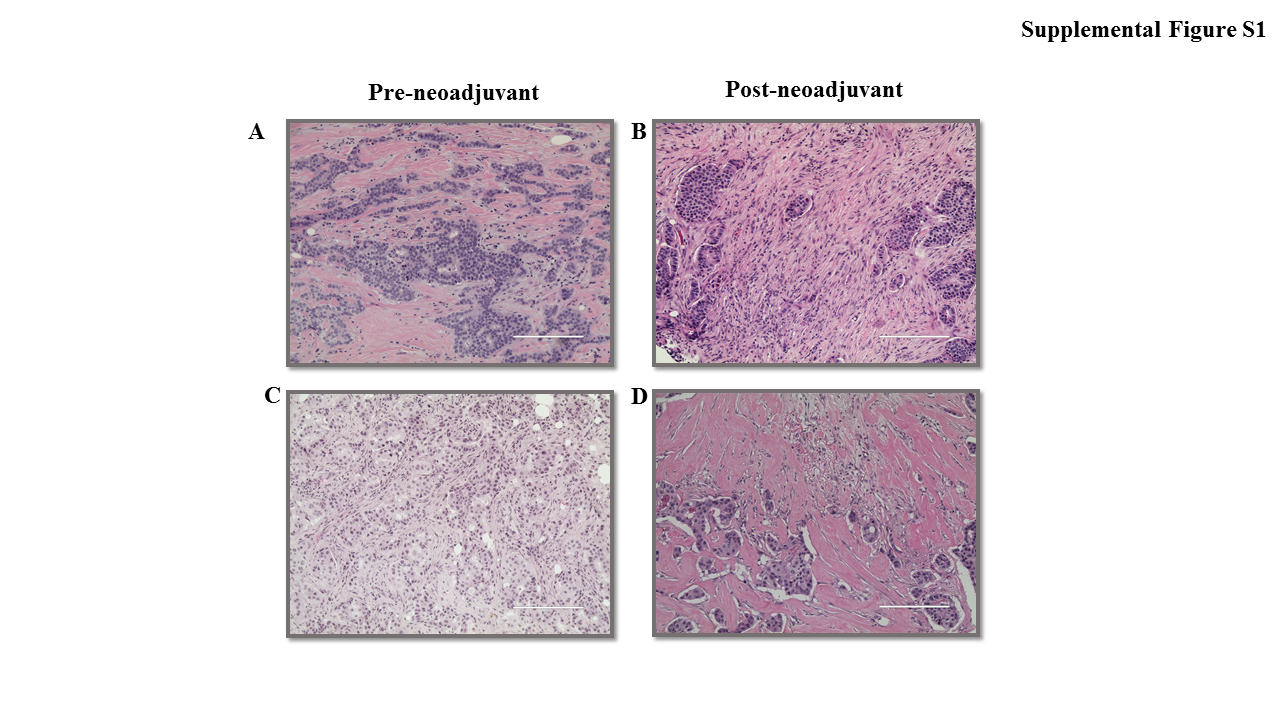

Supplement: Supplementary file 2 — Showing HES of TILs at baseline and post-treatment. A Baseline HES of a case with moderate TIL infiltration at baseline and increased TIL counts following treatment. B Matched post-neoadjuvant HES of the baseline biopsy shown in (A). C Baseline HES of a case that displayed decrease TIL counts following treatment. D. Matched post-neoadjuvant HES of the baseline biopsy shown in (C). 20× Magnification, bar = 200 μm. (TIF 948 kb) [file 13058_2017_884_MOESM2_ESM.tif]

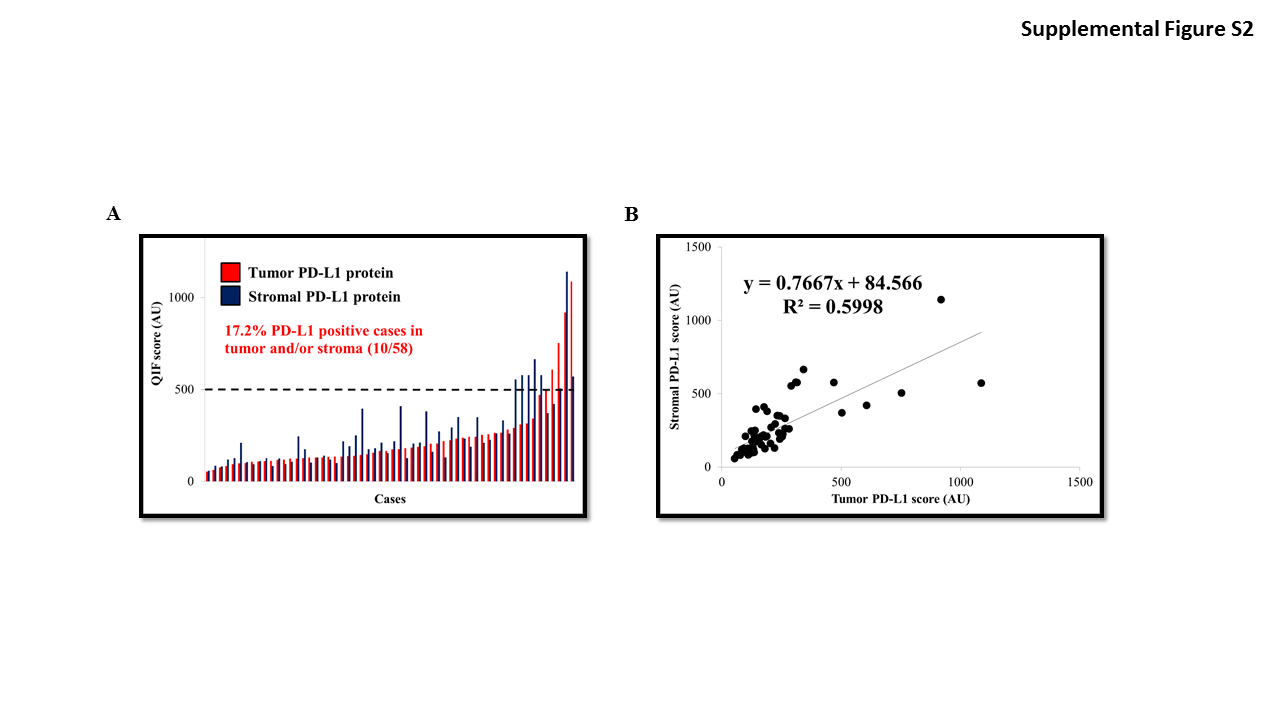

Supplement: Supplementary file 4 — Showing PD-L1 expression in post-neoadjuvant breast cancer specimens. A Distribution of maximal scores of PD-L1 (SP142 antibody) in the tumor (red) and stromal (blue) compartments. The cutoff was set at 500 AQUA units (QIF). B Linear regression of stromal versus tumor PD-L1 AQUA (QIF) scores. (TIF 148 kb) [file 13058_2017_884_MOESM4_ESM.tif]

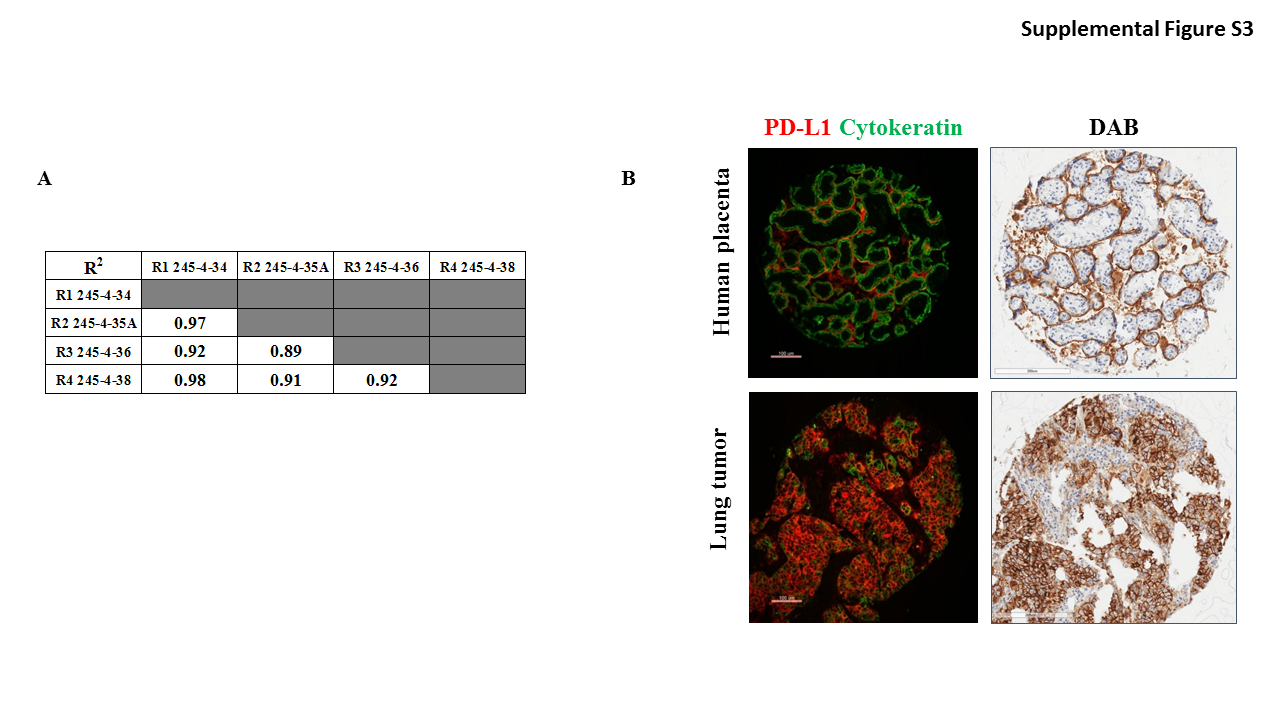

Supplement: Supplementary file 5 — Showing SP142 antibody validation and reproducibility. A Regressions in QIF scores (average) between staining performed in different days in serial sections of a lung cancer TMA (245). B Representative immunostaining for PD-L1 SP142 antibody in control tissues (placenta in upper panel and lung in lower panel) using QIF (left panel; SP142 in Cy5, red and cytokeratin mask in Cy3, green) and conventional IHC staining with DAB (right panel). (TIF 583 kb) [file 13058_2017_884_MOESM5_ESM.tif]
