# Supplementary material for: Effect of neoadjuvant chemotherapy on tumor-infiltrating lymphocytes and PD-L1 expression in breast cancer and its clinical significance
Source: Breast Cancer Res. 2017 Aug 7;19:91. doi: 10.1186/s13058-017-0884-8 (PMC5547502; doi:10.1186/s13058-017-0884-8)
Supplement: Supplementary file 3 — Presenting logistic regression of TIL percentage and PD-L1 scores with survival. (DOCX 14 kb) [file 13058_2017_884_MOESM3_ESM.docx]

| **Table S2. Logistic regression of TILs percentage and PD-L1 scores with survival** | | | | | | | | |  |  |  |  |  |  |  |  |
| --- | --- | --- | --- | --- | --- | --- | --- | --- | --- | --- | --- | --- | --- | --- | --- | --- |
|  | **Per unit change** | | | | **Per change over entire range** | | | |  |  |  |  |  |  |  |  |
|  | **Survival** | **p value** | **Risk ratio** | **95% C.I.** | **Survival** | **p value** | **Risk ratio** | **95% C.I.** |  |  |  |  |  |  |  |  |
| **TILs percentage** |  |  |  |  |  |  |  |  |  |  |  |  |  |  |  |  |
|  | **RFS** | 0.66 | 0.99 | 1.93-1.04 | **RFS** | 0.66 | 0.63 | 0.07-4.49 |  |  |  |  |  |  |  |  |
|  | **OS** | 0.26 | 1.03 | 0.98-1.07 | **OS** | 0.26 | 2.91 | 0.43-17.2 |  |  |  |  |  |  |  |  |
| **PD-L1 Tumor score** |  |  |  |  |  |  |  |  |  |  |  |  |  |  |  |  |
|  | **RFS** | 0.09 | 1 | 1-1.0001 | **RFS** | 0.09 | 0.12 | 0.004-1.31 |  |  |  |  |  |  |  |  |
|  | **OS** | 0.45 | 1 | 1-1.0001 | **OS** | 0.45 | 0.47 | 0.05-2.74 |  |  |  |  |  |  |  |  |
| **PD-L1 Stromal score** |  |  |  |  |  |  |  |  |  |  |  |  |  |  |  |  |
|  | **RFS** | 0.29 | 1 | 1-1.001 | **RFS** | 0.29 | 0.23 | 0.01-3.23 |  |  |  |  |  |  |  |  |
|  | **OS** | 0.62 | 1 | 1-1.001 | **OS** | 0.62 | 0.53 | 0.03-5.85 |  |  |  |  |  |  |  |  |
|  | | | | | | | | |  |  |  |  |  |  |  |  |
